# Impact of biologic therapies on risk of major adverse cardiovascular events in patients with psoriasis: systematic review and meta‐analysis of randomized controlled trials

**DOI:** 10.1111/bjd.14964

**Published:** 2017-03-14

**Authors:** W. Rungapiromnan, Z.Z.N. Yiu, R.B. Warren, C.E.M. Griffiths, D.M. Ashcroft

**Affiliations:** ^1^ Centre for Pharmacoepidemiology and Drug SafetyManchester Pharmacy SchoolUniversity of ManchesterManchesterM13 9PTU.K.; ^2^ Dermatology CentreSalford Royal NHS Foundation TrustUniversity of ManchesterManchester Academic Health Science CentreManchesterU.K.

## Abstract

Concerns have been raised regarding an increased risk of major adverse cardiovascular events (MACEs) (myocardial infarction, cerebrovascular accident or cardiovascular death) in patients treated with anti‐interleukin (IL)‐12/23 agents for moderate‐to‐severe psoriasis. We aimed to examine the risk of MACEs in adult patients with plaque psoriasis that are exposed to biologic therapies via a meta‐analysis of randomized controlled trials (RCTs). Data were obtained from systematic searches in the Cochrane Library, MEDLINE and Embase, U.S. Food and Drug Administration, European Medicines Agency, individual pharmaceutical companies online search platforms and five trials registers (up to 31 March 2016). We selected RCTs reporting adverse events in adults with plaque psoriasis receiving at least one licensed dose of biologic therapy, conventional systematic therapy or placebo. We calculated Peto odds ratios (ORs) with 95% confidence intervals (CIs) and calculated *I* ^2^ statistics to assess heterogeneity. Overall, 38 RCTs involving 18 024 patients were included. No MACEs were observed in 29 studies, while nine RCTs reported 10 patients experiencing MACEs. There was no statistically significant difference in risk of MACEs associated with the use of biologic therapies overall (OR 1·45, 95% CI 0·34–6·24); tumour necrosis factor‐α inhibitors (adalimumab, etanercept and infliximab) (OR 0·67, 95% CI 0·10–4·63); anti‐IL‐17A agents (secukinumab and ixekizumab) (OR 1·00, 95% CI 0·09–11·09) or ustekinumab (OR 4·48, 95% CI 0·24–84·77). No heterogeneity was observed in these comparisons. In conclusion, the limited existing evidence suggests that licensed biologic therapies are not associated with MACEs during the short randomized controlled periods in clinical trials.

## Abstract 2

**What's already known about this topic?**

- The association between biologic therapies and the risk of major adverse cardiovascular events (MACEs) among patients with plaque psoriasis is unclear.

**What does this study add?**

- This is the largest meta‐analysis to examine the risk of MACEs and biologic therapies; it includes 38 randomized controlled trials (18 024 patients with psoriasis) and considers ixekizumab, which has only recently been licensed for use in patients with plaque psoriasis.
- The results of this systematic review and meta‐analysis of randomized controlled trials show there was no significant difference in risk of MACEs in patients with plaque psoriasis who were exposed to biologic therapies.

Linked Comment: Piaserico. Br J Dermatol 2017; **176**:849–850

Several observational studies have suggested that patients with severe psoriasis and psoriatic arthritis (PsA) have a higher risk of cardiovascular events such as myocardial infarction (MI), stroke and cardiovascular death.^1^, ^2^, ^3^, ^4^ It is debated whether this represents a causal association or a predisposition due to the underlying risk factors exhibited by patients with severe psoriasis,^5^, ^6^, ^7^ but there is a hypothesis postulating that the inflammatory cascade activated in patients with severe psoriasis may contribute to the development of atherosclerosis. Thus, medications for psoriasis such as biologic therapies, which have anti‐inflammatory effects, could theoretically improve atherosclerosis and therefore modulate the risk of development of cardiovascular disease.^8^, ^9^, ^10^, ^11^, ^12^

Biologic therapies for the treatment of moderate‐severe plaque psoriasis include tumour necrosis factor‐α inhibitors (TNFi), such as infliximab, etanercept and adalimumab; an inhibitor of the p40 subunit common to interleukin (IL)12 and IL23, ustekinumab; and inhibitors of IL‐17A, secukinumab and ixekizumab. It is currently unclear whether any of these therapies could alter the risk of development of cardiovascular disease. However, a number of major adverse cardiovascular events (MACEs) (MI, cerebrovascular accident or cardiovascular death) were observed in psoriasis patients receiving briakinumab, another IL‐12/23 inhibitor, in randomized controlled trials (RCTs), and this has raised concern regarding whether IL‐12/23 inhibitors could be associated with an increased risk of cardiovascular disease.^13^, ^14^ This directly led to the discontinuation of the development programme of briakinumab.^15^ Despite the approval and licensing of several biologic therapies for the treatment of psoriasis by the U.S. Food and Drug Administration (FDA) and the European Medicines Agency (EMA) in the last decade, the cardiovascular safety profile of these medicines is not well established. The aim of this systematic review of RCTs was to examine whether or not there is any association between currently licensed biologic therapies and risk of MACEs in adult patients with plaque psoriasis.

## Methods

A systematic review and meta‐analysis was conducted and reported in line with the Preferred Reporting Items for Systematic Reviews and Meta‐Analyses (PRISMA) statement.^16^

### Eligibility criteria

We included RCTs that reported adverse events (AEs) in adult patients with plaque psoriasis receiving at least one licensed dose of biologic therapy compared with conventional systematic therapy or placebo/no treatment during the randomized controlled phase. The doses of biologic therapies and conventional systemic therapies assigned had to be approved by the U.S. FDA, the EMA or any European country. The outcomes of interest were MACEs [MI, cerebrovascular accident (including ischaemic and haemorrhagic strokes) or cardiovascular death].

### Data sources and search strategy

The Cochrane Library, MEDLINE and Embase were independently searched without language restrictions from their inception dates to 31 March 2016. The search term sets, which consisted of psoriasis, biologic therapies (individual drug names, trade names and drug classes) and study design, were tailored for each database. An example search strategy is provided in Appendix S1 (see Supporting Information). MEDLINE and Embase databases were searched using all search term sets while the Cochrane Library was searched using only search term sets covering psoriasis and biologic therapies. The Cochrane handbook for systematic reviews of interventions recommends that study design should not be used as a search term set to identify RCTs in the Cochrane Library (unlike MEDLINE or Embase).^17^ Both MeSH and free text terms were used to identify relevant trials. In addition, the U.S. FDA, EMA, five trial registries [the U.S. National Institutes of Health Ongoing Trials Register (www.clinicaltrials.gov); the EU Clinical Trials Register (www.clinicaltrialsregister.eu/); the World Health Organization (WHO) International Clinical Trials Registry Platform (http://apps.who.int/trialsearch/); the Australian and New Zealand Clinical Trials Registry (www.anzctr.org.au); and the International Standard Randomised Controlled Trial Number (ISRCTN) registry (www.isrctn.com)] and pharmaceutical company websites [AbbVie marketing Humira^®^ (adalimumab), Pfizer marketing Enbrel^®^ (etanercept), Janssen and Merck marketing Remicade^®^ (infliximab), Janssen marketing Stelara^®^ (ustekinumab), Eli Lilly and Company marketing Taltz^®^ (ixekizumab), and Novartis Pharmaceutical Corporation marketing Cosentyx^®^ (secukinumab)] were searched for additional details of clinical trials. Furthermore, we screened the reference lists of all included studies to determine whether they mentioned any other eligible trials.

### Study process

All abstracts and full‐text articles were read by one investigator (W.R.) in order to screen for the relevant trials. Two investigators (W.R. and Z.Z.N.Y.) double extracted information from eligible RCTs. Three additional authors (D.M.A., C.E.M.G. and R.B.W.) provided advice on the included studies in case any decision was unclear.

### Data extraction and quality assessment

Data relating to the relevant trial comparisons (biologic therapies, conventional systemic therapies, placebo or no treatment) were extracted that included information on study characteristics (number of study sites, blinding, length of the randomized controlled phase and rate of missing patient data (defined as percentage of patients withdrawing during the study period or excluded from the analysis)); patient characteristics [age, sex, history of PsA, weight, duration of psoriasis, Psoriasis Area and Severity Index (PASI) score, and percentage of body surface area (BSA) affected by psoriasis]; interventions (regimens of biologic therapies, conventional systemic therapies and placebo) and the numbers of participants receiving at least one dose of study drug/placebo/no treatment and separate AEs [MI, cerebrovascular accident (ischaemic and haemorrhagic strokes) and cardiovascular death] or MACEs in each intervention group. If the RCTs did not report the number of separate AEs or MACEs, they were recorded as zero events.

For extension RCTs in which treatment assignments were switched (for instance, patients who were initially treated with placebo switched to a biologic therapy), only MACEs before that point were documented. For multiple reports on the same RCT, all data were collated and aligned to a single RCT. If MACEs were reported at multiple follow‐up points, data from the longest randomized follow‐up were selected provided there was a continuation of the control arm. The overall number of MACEs during the randomized controlled phase in the treatment and control groups of the individual RCTs was extracted for patients who received at least one dose of study agent or placebo or did not receive any treatment.

The Cochrane quality assessment tool for RCTs^18^ was used for assessing risk of bias. Eight domains including sequence generation, allocation concealment, blinding of participants, personnel and outcome assessors, incomplete outcome data (defined as missing outcome data owing to patients dropping out during the study period or excluded from the analysis), selective outcome reporting, adjudication of MACEs and baseline imbalance were considered.

### Data analysis

Extracted data were combined for the meta‐analysis using Review Manager (RevMan) 5·3 (The Nordic Cochrane Centre, The Cochrane Collaboration, Copenhagen, Denmark). Peto odd ratios (ORs) were calculated as an effect measure to quantify the risk of MACEs in patients receiving biologic therapies compared with placebo/no treatment or the same biologic with different dosing. The Peto OR has been reported to perform better than other meta‐analytical methods for rare event rates (lower than 1%).^19^ There were six main comparisons, which included: (i) any biologic therapies (TNFi, anti‐IL‐17A agents and anti‐IL‐12/23 agent) vs. placebo/no treatment; (ii) TNFi vs. placebo; (iii) anti‐IL‐17A agents (secukinumab and ixekizumab) vs. placebo; (iv) anti‐IL‐12/23 agent (ustekinumab) vs. placebo; (v) ustekinumab 45 mg vs. 90 mg; and (vi) secukinumab 150 mg vs. 300 mg. In the first four comparisons, all licensed doses of biologic therapies were considered. A sensitivity analysis was also undertaken using the Mantel–Haenszel risk difference (RD) to explore whether analysis methods had influence on the results of the comparisons. This method (unlike the Peto OR) does not exclude RCTs without MACEs in both comparison groups.^19^ Heterogeneity between studies was assessed using the χ^2^‐test (*P* < 0·05 was considered statistically significant) and *I* ^2^ statistics (significant heterogeneity, *I* ^2^ > 50%; insignificant heterogeneity, *I* ^2^ < 40%). Funnel plot analysis was used for detection of potential publication bias.

## Results

### Study selection

**Figure 1:**
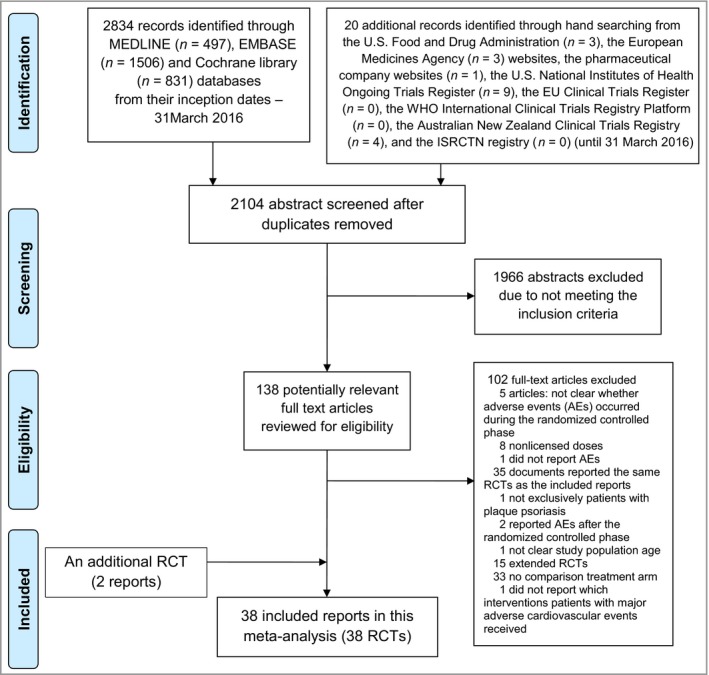
PRISMA flowchart of included randomized controlled trials (RCTs).

In all, 38 RCTs (identified in 38 reports)^20^, ^21^, ^22^, ^23^, ^24^, ^25^, ^26^, ^27^, ^28^, ^29^, ^30^, ^31^, ^32^, ^33^, ^34^, ^35^, ^36^, ^37^, ^38^, ^39^, ^40^, ^41^, ^42^, ^43^, ^44^, ^45^, ^46^, ^47^, ^48^, ^49^, ^50^, ^51^, ^52^, ^53^, ^54^, ^55^, ^56^, ^57^ met the eligibility criteria and were included, as shown in Figure 1. These trials involved a total of 18 024 patients with plaque psoriasis. The 38 RCTs were conducted across a range of 1–231 (median 47) study sites. Thirty‐five RCTs (92·1%) were double‐blind studies. The length of the randomized controlled phase ranged from 10 to 30 (median 12) weeks. The included studies involved from 20 to 1303 patients with plaque psoriasis, with the percentage of male patients ranging from 53% to 90%, the percentage with PsA from 3% to 37%, mean age range 39·2–55·7 years, mean duration of psoriasis range 11·9–21·5 years, mean PASI range 11·5–30·3 (Table S1; see Supporting Information).

Eighteen RCTs compared TNFi (four adalimumab,^20^, ^21^, ^22^, ^23^, ^24^ nine etanercept,^26^, ^27^, ^28^, ^29^, ^30^, ^31^, ^32^, ^34^, ^35^ five infliximab^36^, ^37^, ^38^, ^39^, ^40^) with placebo, with three studies reporting MACEs; four RCTs compared ustekinumab (anti‐IL‐12/23) with placebo^46^, ^47^, ^48^ with no MACEs reported. One RCT compared ixekizumab with placebo without MACEs reported.^56^, ^57^ Six RCTs compared different dose regimens of ustekinumab (three RCTs)^49^, ^50^, ^51^ or secukinumab (anti‐IL‐17A, three RCTs)^43^, ^44^, ^45^ with placebo, with four MACEs reported from three RCTs. One RCT compared ustekinumab 45 mg and 90 mg with etanercept but no MACEs were observed.^52^ Secukinumab 150 mg was compared with 300 mg in one RCT and one patient experienced a MACE in the 300‐mg dose group.^42^ Etanercept (TNFi) was compared with ustekinumab,^53^ secukinumab,^43^ ixekizumab^33^ and placebo/no treatment in four RCTs but only two of them reported two MACEs. One RCT compared adalimumab (TNFi) with placebo and methotrexate (MTX),^25^ one RCT compared adalimumab with MTX^54^ and one RCT compared infliximab (TNFi) with MTX;^41^ no patients in these studies experienced a MACE (Table 1). The overall MACE rates were 0·06% (*n *= 8) for any biologic therapies (total patients 12 596), 0·05% (*n *= 3) for TNFi (total patients 6216), 0·09% (*n *= 3) for anti‐IL‐17A agents (secukinumab and ixekizumab) (total patients 3514), 0·07% (*n *= 2) for ustekinumab (total patients 2866), 0·04% *(n *= 2) for placebo (total patients 5092) and 0% (*n *= 0) for MTX (total patients 336). Seventeen RCTs reported the outcomes using an aggregate MACE definition (this included a study by Papp *et al*. 2008,^49^ which used the term ‘cardiovascular events’ instead of MACEs but its definition was the same as the definition of MACEs in our manuscript) while 21 RCTs reported AEs separately.

**Table 1:** Rates of major adverse cardiovascular events (MACEs) in included randomized controlled trials

First author	Interventions	Number of participants receiving treatment	MACEs	Randomized controlled phase (weeks)
**Adalimumab vs. placebo**				
Menter 2008 (REVEAL)^20^	Adalimumab 80 mg SC at week 0 followed by 40 mg SC every other week starting at week 1	814	0	16
	Placebo at week 0 then every other week starting at week 1	398	0	
Maari 2014^21^	Adalimumab 80 mg followed by 40 mg at week 1 and then 40 mg every other week for 7 weeks	10	0	12
	Placebo for 7 weeks	10	0	
Gordon 2015 (X‐PLORE)^22^	Adalimumab 80 mg SC at week 0 and then 40 mg every other week starting at week 1	43	0	16
	Placebo SC	42	0	
AbbVie 2015, NCT01646073, clinicaltrials.gov^23^	Adalimumab 80 mg SC at week followed by 40 mg SC every other week starting at week 1^24^	338	1	12
	Placebo at week 0 and every other week starting at week 1^24^	87	0	
**Adalimumab vs. methotrexate**				
Goldminz 2015^54^	Adalimumab 80 mg SC at week 0 followed by 40 mg SC every other week	15	0	16
	Methotrexate 7·5–25 mg per week orally	15	0	
**Adalimumab vs. methotrexate vs. placebo**				
Saurat 2008 (CHAMPION)^25^	Adalimumab 80 mg SC at week 0 followed by 40 mg SC every other week starting at week 1	107	0	16
	Methotrexate 7·5–25 mg per week orally	110	0	
	Placebo	53	0	
**Etanercept vs. placebo**				
Gottlieb 2003^26^	Etanercept 25 mg SC twice weekly	57	0	24
	Placebo SC twice weekly	55	1	
Tyring 2006^27^	Etanercept 50 mg SC twice weekly	312	0	12
	Placebo SC twice weekly	306	0	
van de Kerkhof 2008^28^	Etanercept 50 mg SC weekly	96	0	12
	Placebo SC weekly	46	0	
Gottlieb 2011^29^	Etanercept 50 mg SC twice weekly week 0–11	141	0	12
	Placebo SC matching active treatment	68	0	
Strober 2011^30^	Etanercept 50 mg SC twice weekly week 0–11	139	0	12
	Placebo SC matching active treatment	72	0	
Bagel 2012^31^	Etanercept 50 mg SC twice weekly	59	0	12
	Placebo SC twice weekly	62	0	
Bachelez 2015^32^	Etanercept 50 mg SC twice weekly	335	1	12
	Placebo	107	0	
**Etanercept (different strengths) vs. placebo**				
Leonardi 2003^34^	Etanercept 25 mg SC weekly	160	0	12
	Etanercept 25 mg SC twice weekly	162	0	
	Etanercept 50 mg SC twice weekly	164	0	
	Placebo	166	0	
Papp 2005^35^	Etanercept 25 mg SC twice weekly	196	0	12
	Etanercept 50 mg SC twice weekly	194	0	
	Placebo SC twice weekly	193	0	
**Etanercept vs. ixekizumab vs. placebo**				
Griffiths 2015 (UNCOVER‐2)^33^	Etanercept 50 mg SC twice weekly	357	1	12
	Ixekizumab 160 mg SC week 0 then 80 mg SC every 2 weeks	350	0	
	Placebo	167	0	
Griffiths 2015 (UNCOVER‐3)^33^	Etanercept 50 mg SC twice weekly	382	0	12
	Ixekizumab 160 mg SC week 0 then 80 mg SC every 2 weeks	384	0	
	Placebo	193	1	
**Infliximab vs. placebo**				
Chaudhari 2001^36^	Infliximab 5 mg ml^−1^ IV at week 0, 2 and 6	11	0	10
	Placebo IV at week 0, 2 and 6	11	0	
Gottlieb 2004 (SPIRIT)^37^	Infliximab 5 mg kg^−1^ IV infusion at week 0, 2 and 6. At week 26, if patients had a static Physician's Global Assessment of moderate to severe disease, they received a single additional IV infusion of infliximab 5 mg kg^−1^	99	0	30
	Placebo IV infusion at week 0, 2 and 6. At week 26, if patients had a static Physician's Global Assessment of moderate to severe disease, they received a single additional IV infusion of placebo	51	0	
Reich 2005 (EXPRESS)^38^	Infliximab 5 mg kg^−1^ IV at week 0, 2 and 6 and every 8 weeks	298	0	24
	Placebo at week 0, 2, 6, 14 and 22	76	0	
Menter 2007 (EXPRESS II)^39^	Infliximab 5 mg kg^−1^ infusion at week 0, 2 and 6	314	0	14
	Placebo infusion at week 0, 2 and 6	207	0	
Yang 2012^40^	Infliximab 5 mg kg^−1^ IV drip infusion week 0, 2 and 6	84	0	10
	Placebo IV drip infusion week 0, 2 and 6	45	0	
**Infliximab vs. methotrexate**				
Barker 2011 (RESTORE1)^41^	Infliximab 5 mg kg^−1^ at week 0, 2, 6, 14 and 22	649	0	16
	Methotrexate 15 mg weekly with a dose increase to 20 mg weekly at week 6 if Psoriasis Area and Severity Index response < 25%	211	0	
**Ixekizumab vs. placebo**				
Gordon 2016 (UNCOVER‐1)^57^	Ixekizumab 160 mg SC week 0 then 80 mg SC every 2 weeks	433	0	12
	Placebo SC week 0 then every 2 weeks	431	0	
**Secukinumab 150 mg vs. secukinumab 300 mg**				
Mrowietz 2015 (SCULPTURE)^42^	Secukinumab 150 mg SC at week 0, 1, 2, 3, 4 and 8	482	0	12
	Secukinumab 300 mg SC at week 0, 1, 2, 3, 4 and 8	483	1	
**Secukinumab 150 mg vs. secukinumab 300 mg vs. placebo**				
Langley 2014 (ERASURE)^43^	Secukinumab 150 mg SC at week 0, 1, 2, 3, 4 then every 4 weeks	245	0	12
	Secukinumab 300 mg SC at week 0, 1, 2, 3, 4 then every 4 weeks	245	0	
	Placebo at week 0, 1, 2, 3, 4 then every 4 weeks	247	0	
Blauvelt 2015 (FEATURE)^44^	Secukinumab 150 mg SC week 0, 1, 2, 3, 4 and 8	59	0	12
	Secukinumab 300 mg SC week 0, 1, 2, 3, 4 and 8	59	2	
	Placebo SC week 0, 1, 2, 3, 4 and 8	59	0	
Paul 2015 (JUNCTURE)^45^	Secukinumab 150 mg SC week 0, 1, 2, 3, 4 and 8	61	0	12
	Secukinumab 300 mg SC week 0, 1, 2, 3, 4 and 8	60	0	
	Placebo SC week 0, 1, 2, 3, 4 and 8	61	0	
**Ustekinumab vs. placebo**				
Tsai 2011 (PEARL)^46^	Ustekinumab 45 mg SC at week 0 and 4	61	0	12
	Placebo SC at week 0 and 4	60	0	
Zhu 2013 (LOTUS)^47^	Ustekinumab 45 mg SC at week 0 and 4	160	0	12
	Placebo SC at week 0 and 4	161	0	
Lebwohl 2015 (AMAGINE 2)^48^	Ustekinumab SC (45 mg for patients with a body weight ≤ 100 kg and 90 mg for patients with a body weight > 100 kg) on day 1 and week 4	300	0	12
	Placebo	309	0	
Lebwohl 2015 (AMAGINE 3)^48^	Ustekinumab SC (45 mg for patients with a body weight ≤ 100 kg and 90 mg for patients with a body weight > 100 kg) on day 1 and week 4	313	0	12
	Placebo	315	0	
**Ustekinumab 45 mg vs. ustekinumab 90 mg vs. placebo**				
Leonardi 2008 (PHOENIX 1)^51^	Ustekinumab 45 mg SC at week 0 and 4	255	1	12
	Ustekinumab 90 mg SC at week 0 and 4	255	0	
	Placebo at week 0 and 4	255	0	
Papp 2008 (PHOENIX 2)^49^	Ustekinumab 45 mg SC at week 0 and 4	409	0	12
	Ustekinumab 90 mg SC at week 0 and 4	411	1	
	Placebo	410	0	
Igarashi 2012^50^	Ustekinumab 45 mg SC at week 0 and 4	64	0	12
	Ustekinumab 90 mg SC at week 0 and 4	62	0	
	Placebo SC at week 0 and 4	32	0	
**Etanercept vs. ustekinumab 45 mg vs. ustekinumab 90 mg**				
Griffiths 2010 (ACCEPT)^52^	Etanercept 50 mg SC twice weekly	347	0	12
	Ustekinumab 45 mg SC at week 0 and 4	209	0	
	Ustekinumab 90 mg SC at week 0 and 4	347	0	
**Etanercept vs. ustekinumab vs. no treatment**				
Merck Sharp & Dohme 2015, NCT01276847, clinicaltrials.gov^53^	Etanercept 50 mg SC twice weekly for 12 weeks then SC weekly for 4 weeks	10	0	16
	Ustekinumab 45 mg SC for participants weighing ≤ 100 kg, and ustekinumab 90 mg SC for participants weighing > 100 kg on day 1, and weeks 4 and 16	20	0	
	No treatment	10	0	
**Etanercept vs. secukinumab 150 mg vs. seckinumab 300 mg vs. placebo**				
Langley 2014 (FIXTURE)^43^	Etanercept 50 mg SC twice weekly	323	0	12
	Secukinumab 150 mg SC weekly week 0, 1, 2, 3, 4 then every 4 weeks	327	0	
	Secukinumab 300 mg SC weekly week 0, 1, 2, 3, 4 then every 4 weeks	326	0	
	Placebo at weeks corresponding to etanercept and secukinumab regimens	327	0	

IV, intravenous; SC, subcutaneous.

### Meta‐analysis

**Figure 2:**
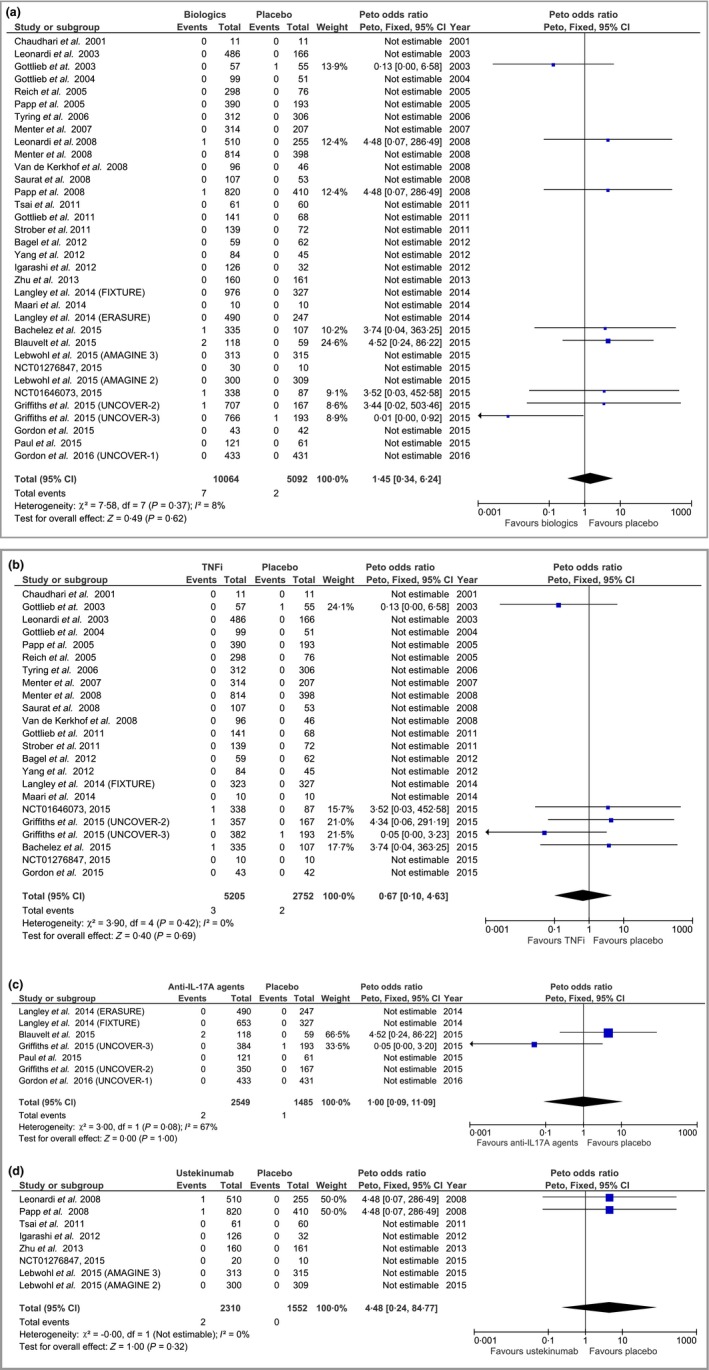
Peto odds ratio (OR) of major adverse cardiovascular events in patients treated with (a) biologic therapies vs. placebo; (b) tumour necrosis factor‐α inhibitors (TNFi) vs. placebo, (c) anti‐interleukin‐(IL)‐17A agents vs. placebo; and (d) ustekinumab vs. placebo. CI, confidence interval; df, degrees of freedom.

Patients in 27 RCTs^20^, ^21^, ^22^, ^25^, ^27^, ^28^, ^29^, ^30^, ^31^, ^34^, ^35^, ^36^, ^37^, ^38^, ^39^, ^40^, ^43^, ^45^, ^46^, ^47^, ^48^, ^50^, ^52^, ^53^, ^57^ did not experience MACEs while exposed to any interventions but 10 MACEs were observed during the randomized controlled phase of nine studies.^23^, ^26^, ^32^, ^33^, ^42^, ^44^, ^49^, ^51^ Overall, the pooled analysis of these nine trials found that there was no statistically significant difference in the risk of MACEs when comparing biologic therapies with placebo (pooled OR 1·45, 95% CI 0·34–6·24, *P *= 0·62), as shown in Figure 2a. We found very low levels of heterogeneity between the included RCTs (χ^2^ **= 7·58; degrees of freedom* *= 7; *P *= 0·37; *I* ^2^ **= 8%).

Considered separately, there was also no statistically significant difference for patients receiving TNFi (adalimumab, etanercept and infliximab) anti‐IL‐17A agents (secukinumab and ixekizumab), or ustekinumab. The corresponding pooled ORs were 0·67, 95% CI 0·10–4·63, *P *= 0·69 for TNFi (Fig. 2b); 1·00, 95% CI 0·09–11·09, *P *= 1·00 for anti‐IL‐17A agents (Fig. 2c); and 4·48, 95% CI 0·24–84·77, *P *= 0·32 for ustekinumab (Fig. 2d). Comparing ustekinumab 45 mg against 90 mg and secukinumab 150 mg against 300 mg, the ORs suggest there were no statistically significant differences in the risk of MACEs (OR 1·00, 95% CI 0·06–16·03, *P *= 1·00 in four ustekinumab trials and OR 0·13, 95% CI 0·01–1·30, *P *= 0·08 in five secukinumab trials). The sensitivity analyses using the Mantel–Haenszel risk difference found similar results for all comparisons (Fig. S1; see Supporting Information).

### Risk of bias assessment

Our risk of bias assessment found that 28 RCTs (74%; low risk of bias) adequately reported generation of the random sequence, 27 RCTs (71%) adequately concealed allocation; 22 RCTs (58%) and 21 RCTs (55%) blinded patients and personnel, and outcome assessors, respectively. Incomplete outcome data were well balanced in 33 RCTs (87%). Fifteen RCTs (40%) explicitly stated that cardiovascular events were monitored and/or these outcomes were reported. Only 10 RCTs (26%) had a committee for adjudicating suspected MACEs. Among 36 RCTs (95%), patient characteristics in all intervention groups were well balanced (Table S2; see Supporting Information).

Funnel plot analysis using the Peto method was used for assessing potential publication bias; visual inspection of the funnel plot for the outcomes in TNFi studies showed no evidence of publication bias. For the Mantel–Haenszel fixed‐effect method, funnel plot analysis also showed no evidence of publication bias in all comparisons.

## Discussion

In this meta‐analysis of RCTs, we found that there was no statistically significant difference in the risk of MACEs in patients with plaque psoriasis exposed to biologic therapies (adalimumab, etanercept, infliximab, ustekinumab, secukinumab and ixekizumab) used at the licensed doses compared with placebo. Moreover, no difference in risk was also found for comparisons between different licensed doses of ustekinumab (45 mg vs. 90 mg) or secukinumab (150 mg vs. 300 mg).

Two earlier meta‐analyses of RCTs have examined the risk of MACEs and biologic therapies for the treatment of psoriasis. The first included 22 trials and reported that TNFi (adalimumab, etanercept and infliximab) and anti‐IL‐12/23 agents (ustekinumab and briakinumab) were not associated with an increased risk of MACEs.^58^ This meta‐analysis used a Mantel–Haenszel fixed effect model to examine absolute risk difference, which is generally considered a less appropriate method for detecting rare events (lower than 1%).^19^ The second meta‐analysis included nine trials to examine the association between MACEs and anti‐IL‐12/23 agents (ustekinumab and briakinumab).^59^ The results of this analysis suggested that anti‐IL‐12/23 agents were significantly associated with an increased risk of MACEs. In our meta‐analysis, we did not include briakinumab as this has not been licensed for use by the regulatory agencies. However, we did include newer licensed biologic therapies (secukinumab and ixekizumab) in our analysis. One important limitation of the earlier meta‐analyses is that they included patients treated with both nonlicensed and licensed doses of biologic therapies, while our meta‐analysis has focused only on those patients receiving biologic therapies at licensed dose regimens.

Nonetheless, we were faced with several important limitations that should be considered when interpreting the findings of our meta‐analysis. Firstly, the primary aim of all the included trials was to examine efficacy and only 10 trials explicitly provided a definition of MACEs and established a committee for adjudicating suspected cases. Most of the included trials had a relatively small sample size and a short duration of the randomized controlled phase of treatment (ranging from 10 to 30 weeks). These factors would impact on the power of the included studies to detect a change in risk of MACEs and this uncertainty was reflected by the wide CIs surrounding some of our risk estimates. For instance, ustekinumab has been suggested to increase the risk of MACEs during the initial stage of therapy because of temporary increases in inflammatory mediators.^60^ A phase 2 study showed that serum levels of IL‐12/23 p40, which is proatherogenic, in patients receiving ustekinumab dramatically increased at week 12 and decreased to a little above baseline levels by week 32.^61^ Thus, assessment of the potential association requires continued surveillance of emerging trial data. In cardiovascular research, it is also well established to use composite outcomes including MACE to detect rare events; this will increase the power to detect clinically important differences in event rates.^62^ Ideally, the recent calls to facilitate the sharing of clinical trial data will also provide new opportunities to examine individual patient‐level data from RCTs thereby enabling more robust time‐to‐event meta‐analysis to be performed.^63^

The majority of the included studies were phase 3 trials, which tend to enrol patients with fewer comorbidities than those seen in routine clinical practice and also exclude elderly patients, who are at increased risk of MACEs. Thus, the background risk for both the exposed and nonexposed groups is likely to be lower, which may limit the generalizability of the findings.

Two cohort studies have recently reported that biologic therapies were not associated with the risk of MACEs in patients with psoriasis.^64^, ^65^ The first study, using a Danish nationwide cohort, analysed the association with biologic therapies; the results suggested that TNFi were associated with a significantly decreased risk of MACEs but there was no difference in risk associated with ustekinumab. This study did not examine the TNFi separately, which is likely to be due to the relatively small numbers of patients receiving TNFi (*n *= 959) and ustekinumab (*n *= 178). In addition, the study did not adjust for important confounders such as body mass index or smoking status in the analysis. The second study recruited 12 095 participants with psoriasis receiving biologic therapies and nonbiologic systemic therapies (such as MTX, or ciclosporin). In this study, 96·5%, 85·6% and 85·8% of patients in the infliximab, ustekinumab, and other biologic therapies groups, respectively, received at least one biologic therapy before study entry. Thus, the MACEs that occurred during the study period might not be solely related to their treatment group and a ‘new user’ study design would have been preferable to avoid this risk of bias.^66^ In addition, a number of important cardiovascular risk factors such as diabetes mellitus, hypertension and dyslipidaemia were not adjusted for when assessing the association between MACEs and biologic therapies.

An earlier retrospective cohort study involving 8845 patients with psoriasis examined the association between TNFi (etanercept, infliximab or adalimumab) and risk of MI.^67^ The study reported that TNFi were associated with a significantly reduced risk of MI when compared with topical therapies but this association was not found when comparisons were made with oral systemic agents (ciclosporin, acitretin and MTX) or phototherapy. Comparing TNFi with topical therapies would not be an appropriate comparison because patients treated with topical therapies are at a much earlier stage in the treatment pathway, and those patients with psoriasis who are most likely to experience MI (including fatal events) may do so before being exposed to biologic therapies, which could bias results.^68^

In conclusion, the existing evidence suggests that biologic therapies including TNFi, an anti‐IL‐12/23 agent (ustekiumab) and anti‐IL‐17A agents (secukinumab and ixekizumab) had no significant impact on the risk of MACEs in adult patients with plaque psoriasis over the short term. Moreover, nor did the different licensed dosages of ustekinumab and secukinumab impact on the risk of MACEs. However, these results should be interpreted with caution given the short duration of follow‐up and the characteristics of patients participating in RCTs. Well‐designed observational studies that involve larger numbers of patients and longer durations of treatment exposure reflecting routine clinical practice are required in order to better examine the impact of biologic therapies on the risk of MACEs in patients with psoriasis.

## Supporting information


**Appendix S1.** Search strategy.
**Table S1.** Characteristics of included randomized controlled trials.
**Table S2.** Risk of bias assessment for randomized controlled trials.
**Fig S1.** Mantel–Haenszel risk difference of major adverse cardiovascular events between therapies.

**Video S1.** Author video.
