# Supplementary material for: Impact of biologic therapies on risk of major adverse cardiovascular events in patients with psoriasis: systematic review and meta‐analysis of randomized controlled trials
Source: Br J Dermatol. 2017 Mar 14;176(4):890–901. doi: 10.1111/bjd.14964 (PMC5412670; doi:10.1111/bjd.14964)
Supplement: Supplementary file 1 — Appendix S1. Search strategy. Table S1. Characteristics of included randomized controlled trials. Table S2. Risk of bias assessment for randomized controlled trials. Fig S1. Mantel–Haenszel risk difference of major adverse cardiovascular events between therapies. [file BJD-176-890-s001.docx]

**Supporting Information**

**Appendix S1: Search strategy:** MEDLINE (Ovid)

1 exp Psoriasis/

2 psoriasis$.ti,ab.

3 psoriatic$.ti,ab.

4 1 or 2 or 3

5 exp Biological Products/

6 biologic$ product$.ti,ab.

7 exp Biological Therapy/

8 biologic$ therap$.ti,ab.

9 biologic$ treatment$.ti,ab.

10 biologic$ medicine$.ti,ab.

11 biologic$ medication$.ti,ab.

12 biologic$ agent$.ti,ab.

13 tumo?r necrosis factor inhibitor$.ti,ab.

14 tumo?r necrosis factor alpha inhibitor$.ti,ab.

15 TNF inhibitor$.ti,ab.

16 TNF blocker$.ti,ab.

17 TNFi$.ti,ab.

18 TNF-alpha inhibitor$.ti,ab.

19 anti-TNF treatment$.ti,ab.

20 anti-TNF therap$.ti,ab.

21 anti-TNF alpha therap$.ti,ab.

22 anti-TNF alpha treatment$.ti,ab.

23 Anti-TNF agent$.ti,ab.

24 Anti-TNF-alpha agent$.ti,ab.

25 adalimumab.ti,ab.

26 Humira.ti,ab.

27 etanercept.ti,ab.

28 Enbrel.ti,ab.

29 infliximab.ti,ab.

30 Remicade.ti,ab.

31 exp Antibodies, Monoclonal/

32 monoclonal antibod$.ti,ab.

33 ustekinumab.ti,ab.

34 Stelara.ti,ab.

35 secukinumab.ti,ab.

36 Cosentyx.ti,ab.

37 ixekizumab.ti,ab.

38 Taltz.ti,ab.

39 5 or 6 or 7 or 8 or 9 or 10 or 11 or 12 or 13 or 14 or 15 or 16 or 17 or 18 or 19 or 20 or

21 or 22 or 23 or 24 or 25 or 26 or 27 or 28 or 29 or 30 or 31 or 32 or 33 or 34 or 35 or

36 or 37 or 38

40 "randomized controlled trial".pt.

41 (random$ or placebo$ or single blind$ or double blind$ or triple blind$).ti,ab.

42 (retraction of publication or retracted publication).pt.

43 or/40-42

44 (animals not humans).sh.

45 ((comment or editorial or meta-analysis or practice-guideline or review or letter or

journal correspondence) not "randomized controlled trial").pt.

46 (random sampl$ or random digit$ or random effect$ or random survey or random

regression).ti,ab. not "randomized controlled trial".pt.

47 43 not (44 or 45 or 46)

48 4 and 39 and 47

**Table S1. Characteristics of included randomize controlled trials**

| **Authors, year** | **No. of study sites** | **Masking** | **Randomized controlled-phase (weeks)** | **Interventions during randomized controlled-phase** | **No. of participants receiving treatment** | **Mean age ± SD (median) years** | **Male, *n* (%)** | **History of psoriatic arthritis, *n* (%)** | **Mean weight ± SD (median) kg** | **Mean duration of psoriasis** ± **SD (median) years** | **Mean Psoriasis Area and Severity Index Score ± SD (median)** | **Mean Body Surface Area ± SD (median) (%)** | **Missing outcome data** |
| --- | --- | --- | --- | --- | --- | --- | --- | --- | --- | --- | --- | --- | --- |
| **Adalimumab vs placebo** | | | | | | | | | | | | | |
| Menter et.al., 2008 (REVEAL)^1^ | 81 | Double-blind | 16 | Adalimumab 80 mg subcutaneous (SC.) at week 0 followed by 40 mg SC. every other week (EOW) starting at week 1 | 814 | 44.1 ± 13.2 | 546 (67.1) | 224 (27.5) | 92.3 ± 23.0 | 18.1 ± 11.91 | 19.0 ± 7.08 | 25.8 ± 15.51 | 3.8% |
|  |  |  |  | Placebo at week 0 then EOW starting at week 1 | 398 | 45.4 ± 13.4 | 257 (64.6) | 113 (28.4) | 94.1 ± 23.0 | 18.4 ± 11.94 | 18.8 ± 7.09 | 25.6 ± 14.76 | 10.8% |
| Maari et.al., 2014^2^ | 1 | Double-blind | 12 | Adalimumab 80 mg followed by 40 mg at week 1 and then 40 mg EOW for 7 weeks | 10 | 55.7 ± 11.8 | 9 (90) | Not reported (NR) | 132.0 ± 22.2 | NR | 11.5 ± 6.3 | 12.5 ± 11.0 | 0% |
|  |  |  |  | Placebo for 7 weeks | 10 | 49.0 ± 10.9 | 9 (90) | NR | 135.9 ± 31.5 | NR | 10.4 ± 4.5 | 10.0 ± 5.0 | 0% |
| Gordon et.al., 2015 (X-PLORE)^3^ | 43 | Double-blind | 16 | Adalimumab 80 mg SC. at week 0 and then 40 mg EOW starting at week 1 | 43 | (50.0) | 30 (69.8) | 11 (25.6) | 91.6 ± 19.88 | 19.3 ±12.79 | 20.2 ± 7.58 (17.9) | 26.8 ± 16.80 | 9.3% |
|  |  |  |  | Placebo SC. | 42 | (46.5) | 28 (66.7) | 12 (28.6) | 93.6 ± 22.62 | 18.0 ± 13.30 | 21.8 ± 9.98 (17.3) | 27.5 ± 19.26 | 7.1% |
| AbbVie 2015, NCT01646073, clinicaltrials.gov^4^ | 16 | Double-blind | 12 | Adalimumab 80 mg SC. at week 0 followed by 40 mg SC. EOW starting at week 1^5^ | 338 | 43.1 ± 11.91 | 254 (75.1) | NR | NR | 14.8 ± 10.11 | 28.2 ± 12.00 | 42.6 ± 21.75 | 1.5% |
|  |  |  |  | Placebo SC. at week 0 and EOW starting at week 1^5^ | 87 | 43.8 ± 12.45 | 58 (66.7) | NR | NR | 15.8 ± 10.31 | 25.60 ± 10.98 | 39.3 ± 22.50 | 2.4% |
| **Adalimumab vs methotrexate** | | | | | | | | | | | | | |
| Goldminz et.al., 2015^6^ | 1 | Open-label | 16 | Adalimumab 80 mg SC. at week 0 followed by 40 mg SC. EOW | 15 | 50.5 | 11 (73.3) | 2 (13.3) | NR | 17.3 (1 - 45) | 16.8 | NR | 6.7% |
|  |  |  |  | Methotrexate 7.5 - 25 mg/week orally | 15 | 50.3 | 13 (86.7) | 3 (20.0) | NR | 21.5 (0 - 47) | 15.9 | NR | 0% |
| **Adalimumab vs methotrexate vs placebo** | | | | | | | | | | | | | |
| Saurat et.al., 2008 (CHAMPION)^7^ | 28 | Double- blind | 16 | Adalimumab 80 mg SC. at week 0 followed by 40 mg SC. EOW starting at week 1 | 107 | 42.9 ± 12.6 | 70 (64.8) | 23 (21.3) | 81.7 ± 20.0 | 17.9 ± 10.1 | 20.2 ± 7.5 | 33.6 ± 19.9 | 3.7% |
|  |  |  |  | Methotrexate 7.5 - 25 mg/week orally | 110 | 41.6 ± 12.0 | 73 (66.4) | 19 (17.3) | 83.1 ± 17.5 | 18.9 ± 10.2 | 19.4± 7.4 | 32.4 ± 20.6 | 5.5% |
|  |  |  |  | Placebo | 53 | 40.7 ± 11.4 | 35 (66.0) | 11 (20.8) | 82.6 ± 19.9 | 18.8 ± 8.7 | 19.2 ± 6.9 | 28.4 ± 16.1 | 9.4% |
| **Etanercept vs placebo** | | | | | | | | | | | | | |
| Gottlieb et.at., 2003^8^ | Multicentres | Double-blind | 24 | Etanercept 25 mg SC. twice weekly (BIW) | 57 | 48.2 | 33 (58) | 16 (28.1) | 91.8 | 23 | 17.8 | 30 | 15.8% |
|  |  |  |  | Placebo SC. BIW | 55 | 46.5 | 37 (67) | 19 (34.5) | 90.7 | 20 | 19.5 | 34 | 78.2% |
| Tyring et.al., 2006^9^ | 39 | Double-blind | 12 | Etanercept 50 mg SC. BIW | 312 | 45.8 ± 12.8 | 203 (65.3) | 109 (35.0) | NR | 20.1 ± 12.3 | 18.3 ± 7.6 | 27.2 ± 18.2 | 1.9% |
|  |  |  |  | Placebo SC. BIW | 306 | 45.6 ± 12.1 | 216 (70.4) | 100 (32.6) | NR | 19.7 ± 11.4 | 18.1 ± 7.4 | 27.2 ± 17.2 | 5.5% |
| van de Kerkhof et.al., 2008^10^ | NR | Double-blind | 12 | Etanercept 50 mg SC. once weekly (QW) | 96 | 45.9 ± 12.8 | 59 (61.5) | 15 (15.6) | 83.4 ± 16.0 | 19.3 ± 11.3 | 21.4 ± 9.3 | 26.5 ± 15.0 | 6.3% |
|  |  |  |  | Placebo SC. QW | 46 | 43.6 ± 12.6 | 25 (54.4) | 5 (10.9) | 79.1 ± 20.2 | 17.3 ± 8.2 | 21.0 ± 8.7 | 30.3 ± 17.8 | 21.7% |
| Gottlieb et.al., 2011^11^ | 33 | Double-blind | 12 | Etanercept 50 mg SC. BIW week 0 - 11 | 141 | 43.1 ± 12.5 | 98 (69.5) | 32 (22.7) | 94.5 ± 20.4 | 17.0 ± 12.7 | 19.4 ± 8.0 | 24.1 ± 15.0 | 5.0% |
|  |  |  |  | Placebo SC. matching active treatment | 68 | 44.0 ± 13.6 | 47 (69.1) | 14 (20.6) | 96.5 ± 27.2 | 19.1 ± 13.2 | 18.5 ± 6.9 | 23.8 ± 15.5 | 7.4% |
| Strober et.al., 2011^12^ | 41 | Double-blind | 12 | Etanercept 50 mg SC. BIW week 0 - 11 | 139 | 45.2 ± 14.8 | 85 (61.2) | 46 (33.1) | 96.9 ± 24.9 | 15.2 ± 12.1 | 18.5 ± 6.0 | 24.7 ± 13.9 | 8.6% |
|  |  |  |  | Placebo SC. matching active treatment | 72 | 45.0 ± 13.9 | 46 (63.9) | 15 (20.8) | 92.9 ± 25.2 | 15.5 ± 11.7 | 18.3 ± 6.4 | 22.1 ± 13.4 | 8.3% |
| Bagel et.al., 2012^13^ | NR | Double-blind | 12 | Etanercept 50 mg SC. BIW | 59 | (39) | 33 (53.2) | NR | NR | (17.5) | (15.5) | (15.5) | NR |
|  |  |  |  | Placebo SC. BIW | 62 | (42) | 36 (58.1) | NR | NR | (11.9) | (15.2) | (15.0) | NR |
| Bachelez et.al., 2015^14^ | 122 | Doule blind | 12 | Etanercept 50 mg SC. BIW | 335 | (42.0) | 233 (69.6) | 71 (21.2) | NR | (18.0) | (19.4) | (25.0) | 6.8% |
|  |  |  |  | Placebo | 107 | (46.0) | 71 (66.4) | 26 (21.2) | NR | (17.0) | (19.5) | (26.0) | 12.0% |
| **Etanercept (different strengths) vs placebo** | | | | | | | | | | | | | |
| Leonardi et.al., 2003^15^ | 47 | Double-blind | 12 | Etanercept 25 mg SC. QW | 160 | 44.4 | 118 (74) | NR | NR | 19.3 | 18.2 | 27.7 | NR |
|  |  |  |  | Etanercept 25 mg SC. BIW | 162 | 45.4 | 109 (67) | NR | NR | 18.5 | 18.5 | 28.5 | NR |
|  |  |  |  | Etanercept 50 mg SC. BIW | 164 | 44.8 | 107 (65) | NR | NR | 18.6 | 18.4 | 29.9 | NR |
|  |  |  |  | Placebo | 166 | 45.6 | 105 (63) | NR | NR | 18.4 | 18.3 | 28.8 | NR |
| Papp et.al., 2005^16^ | 50 | Double-blind | 12 | Etanercept 25 mg SC. BIW | 196 | (46.0) | 128 (65.3) | 54 (27.6) | NR | (21.5) | (16.9) | (23.0) | 2.6% |
|  |  |  |  | Etanercept 50 mg SC. BIW | 194 | (44.5) | 130 (67.0) | 50 (25.8) | NR | (18.1) | (16.1) | (25.0) | 2.1% |
|  |  |  |  | Placebo SC. BIW | 193 | (44.0) | 124 (64.2) | 50 (25.9) | NR | (17.5) | (16.0) | (20.0) | 7.8% |
| **Etanercept vs ixekizumab vs placebo** | | | | | | | | | | | | | |
| Griffiths et.al., 2015 (UNCOVER-2)^17^ | 126 | Double-blind | 12 | Etanercept 50 mg SC. BIW | 357 | 45 ± 13 | 236 (66) | NR | NR | 19 ± 12 | 19 ± 7 | 25 ± 16 | 7.0% |
|  |  |  |  | Ixekizumab 160 mg SC. week 0 then 80 mg SC. every 2 weeks | 350 | 45 ± 13 | 221 (63) | NR | NR | 18 ± 12 | 19 ± 7 | 25 ± 16 | 2.6% |
|  |  |  |  | Placebo | 167 | 45 ± 12 | 120 (71) | NR | NR | 19 ± 13 | 21 ± 8 | 27 ± 18 | 6.0% |
| Griffiths et.al., 2015 (UNCOVER-3)^17^ | 126 | Double-blind | 12 | Etanercept 50 mg SC. BIW | 382 | 46 ± 14 | 269 (70) | NR | NR | 18 ± 12 | 21 ± 8 | 28 ± 17 | 3.4% |
|  |  |  |  | Ixekizumab 160 mg SC. week 0 then 80 mg SC. every 2 weeks | 384 | 46 ± 13 | 254 (66) | NR | NR | 18 ± 12 | 21 ± 8 | 28 ± 17 | 5.7% |
|  |  |  |  | Placebo | 193 | 46 ± 12 | 137 (71) | NR | NR | 18 ± 13 | 21 ± 8 | 29 ± 17 | 5.2% |
| **Infliximab vs placebo** | | | | | | | | | | | | | |
| Chaudhari et.al., 2001^18^ | 1 | Double-blind | 10 | Infliximab 5 mg/ml intravenous (IV.) at week 0, 2 and 6 | 11 | 51 ± 14 | 7 (63.6) | NR | 87 ± 20 | NR | 22.1 ± 11.5 | NR | 9.1% |
|  |  |  |  | Placebo IV. at week 0, 2 and 6 | 11 | 45 ± 12 | 8 (72.7) | NR | 85 ± 19 | NR | 20.3 ± 5.5 | NR | 9.1% |
| Gottlieb et.al., 2004 (SPIRIT)^19^ | 24 | Double-blind | 30 | Infliximab 5 mg/kg at IV. infusion at week 0, 2 and 6 At week 26, if patients had a static Physician Global Assessment of moderate to severe disease, they received a single additional IV. Infusion of infliximab 5 mg/kg. | 99 | (44) | 73 (73.7) | 29 (29.3) | NR | (16) | (20) | (25) | 18.2% |
|  |  |  |  | Placebo IV. Infusion at week 0, 2 and 6 At week 26, if patients had a static Physician Global Assessment of moderate to severe disease, they received a single additional IV. infusion of placebo | 51 | (45) | 31 (60.0) | 17 (33.3) | NR | (16) | (18) | (26) | 72.5% |
| Reich et.al., 2005 (EXPRESS)^20^ | 32 | Double-blind | 24 | Infliximab 5 mg/kg IV at week 0, 2 and 6 and every 8 weeks | 298 | 42.6 ± 11.7 | 207 (68.77) | 92 (31) | NR | 19.1 ± 11.0 | 22.9 ± 9.3 | 34.1 ± 19 | 10.6% |
|  |  |  |  | Placebo at week 0, 2, 6, 14 and 22 | 76 | 43.8 ± 12.6 | 61 (79.22) | 22 (29) | NR | 17.3 ± 11.1 | 22.8 ± 8.7 | 33.5 ± 18 | 11.7% |
| Menter et.al., 2007 (EXPRESS II)^21^ | 63 | Double-blind | 14 | Infliximab 5 mg/kg infusion at week 0, 2 and 6 | 314 | 44.5 ± 13.0 (44.0) | 204 (65.0) | 89 (28.3) | 92.2 ± 23.2 (88.8) | 19.1 ± 11.7 (17.9) | 20.4 ± 7.5 (18.6) | 28.7 ± 16.4 (24.0) | 5.4% |
|  |  |  |  | Placebo infusion at week 0, 2 and 6 | 207 | 44.4 ± 12.5 (44.5) | 144 (69.2) | 54 (26.0) | 91.1 ± 22.6 (88.9) | 17.8 ± 10.8 (16.1) | 19.8 ± 7.7 (17.4) | 28.4 ± 17.6 (22.0) | 11.5% |
| Yang et.al., 2012^22^ | 9 | Double-blind | 10 | Infliximab 5 mg/kg IV drip infusion week 0, 2 and 6 | 84 | 39.4 ± 12.3 | 60 (71.4) |  |  | 16.0 ± 10.8 | 23.9 ±10.7 |  | 1.2% |
|  |  |  |  | Placebo IV drip infusion week 0, 2 and 6 | 45 | 40.1 ± 11.1 | 35 (77.8) |  |  | 16.0 ± 8.9 | 25.3 ± 12.7 |  | 2.2% |
| **Infliximab vs methotrexate** | | | | | | | | | | | | | |
| Barker et.al., 2011 (RESTORE1)^23^ | 106 | Open-label | 16 | Inflliximab 5 mg/kg at weeks 0, 2, 6, 14 and 22 | 649 | 44.1 | 438 (67) | NR | 84.5 ± 18.6 | 18.8 ± 11.6 | 21.4 ± 8.0 | 31.9 ± 16.5 | NR |
|  |  |  |  | Methotrexate 15 mg weekly with a dose increase to 20 mg weekly at week 6 if psoriasis area and severity index response < 25 % | 211 | 41.9 | 148 (69) | NR | 83.8 ± 18.2 | 17.0 ± 10.3 | 21.1 ± 7.6 | 31.0 ± 15.0 | NR |
| **Ixekizumab vs placebo** | | | | | | | | | | | | | |
| Gordon et.al., 2016 (UNCOVER-1)^24^ | 110 | Double-blind | 12 | Ixekizumab 160 mg SC. week 0 then 80 mg SC. every 2 weeks | 433 | 45 ± 12 | 291 (67.2) | NA | 92 ± 23^25^ | 20 ± 12 | 20 ± 8 | 28 ± 18 | 4.2% |
|  |  |  |  | Placebo SC week 0 then every 2 weeks | 431 | 46 ± 13 | 303 (70.3) | NA | 92 ± 25^25^ | 20 ± 12 | 20 ± 9 | 27 ± 18 | 5.6% |
| **Secukinumab150 mg vs secukinumab 300 mg** | | | | | | | | | | | | | |
| Mrowietz et.al., 2015 (SCULPTURE)^26^ | 133 | Double-blind | 12 | Secukinumab 150 mg SC. at week 0, 1, 2, 3, 4 and 8 | 482 | 45.3 ± 12.83 | 305 (63.3) | 104 (21.6) | 85.2 ± 22.75 | 17.2 ± 12.71 | 24.0 ± 10.44 | 35.7 ± 21.09 | 3.7% |
|  |  |  |  | Secukinumab 300 mg SC. at week 0, 1, 2, 3, 4 and 8 | 483 | 46.7 ± 12.83 | 333 (68.8) | 94 (19.4) | 85.1 ± 23.20 | 17.4 ± 12.88 | 23.3 ± 9.56 | 33.7 ± 19.56 | 4.1% |
| **Secukinumab150 mg vs secukinumab 300 mg vs placebo** | | | | | | | | | | | | | |
| Langley et.al., 2014 (ERASURE)^27^ | 88 | Double-blind | 12 | Secukinumab 150 mg SC. at week 0, 1, 2, 3, 4 and then every 4 weeks | 245 | 44.9 ± 13.3 | 168 (68.6) | 46 (18.8) | 87.1 ± 22.3 | 17.5 ± 12.0 | 22.3 ± 9.8 | 33.3 ± 19.2 | 6.1% |
|  |  |  |  | Secukinumab 300 mg SC. at week 0, 1, 2, 3, 4 and then every 4 weeks | 245 | 44.9 ± 13.5 | 169 (69.0) | 57 (23.3) | 88.8 ± 24.0 | 17.4 ± 11.1 | 22.5 ± 9.2 | 32.8 ± 19.3 | 2.9% |
|  |  |  |  | Placebo at week 0, 1, 2, 3, 4 and then every 4 weeks | 247 | 45.4 ± 12.6 | 172 (69.4) | 68 (27.4) | 89.7 ± 25.0 | 17.3 ± 12.4 | 21.4 ± 9.1 | 29.7 ± 15.9 | 6.5% |
| Blauvelt et.al., 2015 (FEATURE)^28^ | 32 | Double-blind | 12 | Secukinumab 150 mg SC. week 0, 1, 2, 3, 4 and 8 | 59 | 46.0 ± 15.09 | 40 (67.8) | NR | 93.7 ± 25.64 | 20.4 ± 12.97 | 20.5 ± 8.29 | 30.6 ± 16.65 | 1.7% |
|  |  |  |  | Secukinumab 300 mg SC. week 0, 1, 2, 3, 4 and 8 | 59 | 45.1 ± 12.57 | 38 (64.4) | NR | 92.6 ± 25.94 | 18.0 ± 11.86 | 20.7 ± 7.95 | 33.3 ± 17.98 | 5.1% |
|  |  |  |  | Placebo SC. week 0, 1, 2, 3, 4 and 8 | 59 | 46.5 ± 14.14 | 39 (66.1) | NR | 88.4 ± 21.55 | 20.2 ± 14.22 | 21.1 ± 8.49 | 32.2 ± 17.39 | 5.1% |
| Paul et.al., 2015 (JUNCTURE)^29^ | 38 | Double-blind | 12 | Secukinumab 150 mg SC.week 0, 1, 2, 3, 4 and 8 | 61 | 43.9 ± 14.41 | 41 (67.2) | 16 (26.2) | 93.7 ± 31.71 | 20.6 ± 14.54 | 22.0 ± 8.85 | 30.1 ± 16.66 | 4.9% |
|  |  |  |  | Secukinumab 300 mg SC.week 0, 1, 2, 3, 4 and 8 | 60 | 46.6 ± 14.23 | 46 (76.7) | 14 (23.3) | 91.0 ± 23.13 | 21.0 ± 13.51 | 18.9 ± 6.37 | 26.4 ± 12.77 | 0% |
|  |  |  |  | Placebo SC.week 0, 1, 2, 3, 4 and 8 | 61 | 43.7 ± 12.74 | 38 (62.3) | 12 (19.7) | 90.2 ± 21.16 | 19.86 ± 12.20 | 19.4 ± 6.70 | 25.7 ± 14.70 | 3.3% |
| **Ustekinumab vs placebo** | | | | | | | | | | | | | |
| Tsai et.al., 2011 (PEARL)^30^ | 13 | Double-blind | 12 | Ustekinumab 45 mg SC. at week 0 and 4 | 61 | 40.9 ± 12.7 | 50 (82.0) | 10 (16.4) | 73.1 ± 12.7 | 11.9 ± 7.5 | 25.2 ± 11.9 | 41.8 ± 24.4 | 6.6% |
|  |  |  |  | Placebo SC. at week 0 and 4 | 60 | 40.4 ± 10.1 | 53 (88.3) | 7 (11.7) | 74.6 ± 13.0 | 13.9 ± 7.3 | 22.9 ± 8.6 | 35.8 ± 21.4 | 8.3% |
| Zhu et.al., 2013 (LOTUS)^31^ | 14 | Double-blind | 12 | Ustekinumab 45 mg SC. at week 0 and 4 | 160 | 40.1 ± 12.4 | 125 (78.1) | 14 (8.8) | 69.9 ± 11.9 | 14.6 ± 8.9 | 23.2 ± 9.5 | 35.1 ± 18.5 | 1.9% |
|  |  |  |  | Placebo SC. at week 0 and 4 | 161 | 39.2 ± 12.2 | 123 (75.9) | 14 (8.6) | 70.0 ± 12.6 | 14.2 ± 8.6 | 22.7 ± 9.5 | 35.1 ± 19.6 | 1.9% |
| Lebwohl et.al., 2015 (AMAGINE 2) ^32^ | 142 | Double-blind | 12 | Ustekinumab SC. (45 mg for patients with a body weight ≤ 100 kg and 90 mg for patients with a body weight > 100 kg) on day 1 and week 4 | 300 | 45 ± 13 | 205 (68.3) | 50 (16.7) | 91 ± 24 | 19 ± 13 | 20. ± 8.4 | 27 ± 19 | 3.0% |
|  |  |  |  | Placebo | 309 | 44 ± 13 | 219 (70.9) | 51 (16.5) | 92 ± 23 | 18 ± 12 | 20.4 ± 8.2 | 28 ± 17 | 2.9% |
| Lebwohl et.al., 2015 (AMAGINE 3)^32^ | 142 | Double-blind | 12 | Ustekinumab SC. (45 mg for patients with a body weight ≤ 100 kg and 90 mg for patients with a body weight > 100 kg) on day 1 and week 4 | 313 | 45 ± 13 | 212 (67.7) | 64 (20.4) | 90 ± 22 | 18 ± 12 | 20.1 ± 8.4 | 28 ± 18 | 3.2% |
|  |  |  |  | Placebo | 315 | 44 ± 13 | 208 (66.0) | 59 (18.7) | 89 ± 22 | 18 ± 12 | 20.1 ± 8.7 | 28 ± 17 | 4.4% |
| **Ustekinumab (different strengths) vs placebo** | | | | | | | | | | | | | |
| Leonardi et.al., 2008 (PHOENIX 1)^33^ | 48 | Double-blind | 12 | Ustekinumab 45 mg SC. at week 0 and 4 | 255 | 44.8 ± 12.5 | 175 (68.6) | 74 (29.0) | 93.7 ± 23.8 | 19.7 ± 11.7 | 20.5 ± 8.6 | 27.2 ± 17.5 | 0.4% |
|  |  |  |  | Ustekinumab 90 mg SC. at week 0 and 4 | 255 | 46.2 ± 11.3 | 173 (67.6) | 95 (37.1) | 93.8 ± 23.9 | 19.6 ± 11.1 | 19.7 ± 7.6 | 25.2 ± 15.0 | 4.3% |
|  |  |  |  | Placebo at week 0 and 4 | 255 | 44.8 ± 11.3 | 183 (71.8) | 90 (35.3) | 94.2 ± 3.5 | 20.4 ± 11.7 | 20.4 ± 8.6 | 27.7 ± 17.4 | 4.7% |
| Papp et.al., 2008 (PHOENIX 2)^34^ | 70 | Double-blind | 12 | Ustekinumab 45 mg SC. at week 0 and 4 | 409 | 45.1 ± 12.1 | 283 (69.2) | 107 (26.2) | 90.3 ± 21.0 | 19.3 ± 11.7 | 19.4 ± 6.8 | 25.9 ± 15.5 | 1.5% |
|  |  |  |  | Ustekinumab 90 mg SC. at week 0 and 4 | 411 | 46.6 ± 12.1 | 274 (66.7) | 94 (22.9) | 91.5 ± 21.3 | 20.3 ± 12.3 | 20.1 ± 7.5 | 27.1 ± 17.4 | 2.2% |
|  |  |  |  | Placebo | 410 | 47.0 ± 12.5 | 283 (69.0) | 105 (25.6) | 91.1 ± 21.6 | 20.8 ± 12.2 | 19.4 ± 7.5 | 26.1 ± 17.4 | 4.4% |
| Igarashi et.al, 2012^35^ | 35 | Double-blind | 12 | Ustekinumab 45 mg SC. at week 0, 4 | 64 | 46.6 ± 12.5 (45) ^35,36^ | 53 (82.8) | 6 (9.4) | 73.2 ± 15.4 | 15.8 ± 8.2 | 30.1 ± 12.9 | 47.0 ± 23.7 | 0% |
|  |  |  |  | Ustekinumab 90 mg SC. at week 0, 4 | 62 | 46.8 ± 12.8 (44) ^35,36^ | 47 (75.8) | 7 (11.3) | 71.1 ± 14.0 | 17.3 ± 10.7 | 28.7 ± 11.2 | 46.6 ± 19.7 | 6.5% |
|  |  |  |  | Placebo SC. at week 0, 4 | 32 | 48.5 ± 12.7 (49) ^35,36^ | 26 (83.9) | 1 (3.1) | 71.2 ± 10.9 | 16.0 ± 11.2 | 30.3 ± 11.8 | 49.8 ± 22.5 | 12.5% |
| **Etanercept vs ustekinumab 45 mg vs ustekinumab 90 mg** | | | | | | | | | | | | | |
| Griffiths et.al., 2010 (ACCEPT)^37^ | 67 | Double-blind | 12 | Etanercept 50 mg SC. BIW weekly | 347 | 45.7 ± 13.4 | 246 (70.9) | 95 (27.4) | 90.8 ± 20.9 | 18.8 ± 12.1 | 18.6 ± 6.2 | 23.8 ± 13.9 | 3.2% |
|  |  |  |  | Ustekinumab 45 mg SC. at week 0 and 4 | 209 | 45.1 ± 12.6 | 133 (63.6) | 62 (29.7) | 90.4 ± 21.1 | 18.9 ± 11.8 | 20.5 ± 9.2 | 26.7 ± 17.8 | 3.8% |
|  |  |  |  | Ustekinumab SC. 90 mg SC. at week 0 and 4 | 347 | 44.8 ± 12.3 | 234 (67.4) | 95 (27.4) | 91.0 ± 22.8 | 18.7 ± 11.8 | 19.9 ± 8.4 | 26.1 ± 17.6 | 1.4% |
| **Etanercept vs ustekinumab vs no treatment** | | | | | | | | | | | | | |
| Merck Sharp & Dohme 2015, NCT01276847, clinicaltrials.gov^38^ | NR | Open  label | 16 | Etanercept 50 mg SC. BIW for 12 weeks then SC. QW for 4 weeks | 10 | 39.5 ± 12.5 | 6 (60.0) | NR | NR | NR | NR | NR | 10% |
|  |  |  |  | Ustekinumab 45 mg SC. for participants weighing ≤ 100 kg, and ustekinumab 90 mg SC. for participants weighing > 100 kg on day 1, and weeks 4 and 16 | 20 | 45.7 ± 12.1 | 13 (65.0) | NR | NR | NR | NR | NR | 0% |
|  |  |  |  | No Treatment | 10 | 53.4 ± 13.0 | 9 (90.0) | NR | NR | NR | NR | NR | 0% |
| **Etanercept vs secukinumab 150 mg vs secukinumab 300 mg vs placebo** | | | | | | | | | | | | | |
| Langley et.al., 2014 (FIXTURE)^27^ | 231 | Double-blind | 12 | Etanercept 50 mg SC. BIW | 323 | 43.8 ± 13.0 | 232 (71.2) | 44 (13.5) | 84.6 ± 20.5 | 16.4 ± 12.0 | 23.2 ± 9.8 | 33.6 ± ± 18.0 | 6.4% |
|  |  |  |  | Secukinumab 150 mg SC. QW week 0, 1, 2, 3, 4 and then every 4 weeks | 327 | 45.4 ± 12.9 | 236 (72.2) | 49 (15.0) | 83.6 ± 20.8 | 17.3 ± 12.2 | 23.7 ± 10.5 | 34.5 ± 19.4 | 3.7% |
|  |  |  |  | Secukinumab 300 mg SC. QW week 0, 1, 2, 3, 4 and then every 4 weeks | 326 | 44.5 ± 13.2 | 224 (68.5) | 50 (15.3) | 83.0 ± 21.6 | 15.8 ± 12.3 | 23.9 ± 9.9 | 34.3 ± 19.2 | 4.6% |
|  |  |  |  | Placebo at weeks corresponding to etanercept and secukinumab regimens | 327 | 44.1 ± 12.6 | 237 (72.7) | 49 (15.0) | 82.0 ± 20.4 | 16.6 ± 11.6 | 24.1 ± 10.5 | 35.2 ± 19.1 | 7.7% |

**Table S2. Risk of bias assessment for randomized controlled trials**

| **Authors, year** | **Sequence generation** | **Allocation concealment** | **Blinding of participants and personnel** | **Blinding of outcome assessors** | **Incomplete outcome data** | **Selective outcome reporting** | **Adjudicate of major adverse cardiovascular events** | **Baseline imbalance** |
| --- | --- | --- | --- | --- | --- | --- | --- | --- |
| **Adalimumab vs placebo** | | | | | | | | |
| Menter et.al., 2008 (REVEAL)^1^ | Low | Low | Unclear | Low | Low | Unclear | Unclear | Low |
| Maari et.al., 2014^2^ | Low | Low | Unclear | Unclear | Low | Unclear | Unclear | Low |
| Gordon et.al., 2015 (X-PLORE)^3^ | Unclear | Unclear | High | Unclear | Low | Unclear | Unclear | Low |
| NCT01646073, clinicaltrials.gov, 2015^4^, AbbVie, 2014^5^ | Unclear | Unclear | Unclear | Unclear | Low | Unclear | Unclear | Low |
| **Adalimumab vs methotrexate** | | | | | | | | |
| Goldminz et.al., 2015^6^ | Unclear | Unclear | High | Low | Low | Unclear | Unclear | Low |
| **Adalimumab vs methotrexate vs placebo** | | | | | | | | |
| Saurat et.al., 2008 (CHAMPION)^7^ | Low | Low | Low | Low | Low | Unclear | Unclear | Low |
| **Etanercept vs placebo** | | | | | | | | |
| Gottlieb et.at., 2003^8^ | Low | Low | Low | Low | High | Unclear | Unclear | Low |
| Tyring et.al., 2006^9^ | Low | Low | Unclear | Unclear | Low | Low | Unclear | Low |
| van de Kerkhof et.al., 2008^10^ | Low | Low | Unclear | Unclear | Low | Unclear | Unclear | Low |
| Gottlieb et.al., 2011^11^ | Unclear | Unclear | Unclear | Unclear | Low | Low | Unclear | Low |
| Strober et.al., 2011^12^ | Unclear | Unclear | Unclear | Unclear | Low | Low | Unclear | Low |
| Bagel et.al., 2012^13^ | Low | Low | Unclear | Unclear | Unclear | Unclear | Unclear | Low |
| Bachelez et.al., 2015^14^ | Low | Low | Low | Low | Low | Low | Low | Low |
| **Etanercept (different strengths) vs placebo** | | | | | | | | |
| Leonardi et.al., 2003^15^ | Low | Low | Low | Low | Unclear | Unclear | Unclear | Low |
| Papp et.al., 2005^16^ | Low | Low | Low | Low | Low | Unclear | Unclear | Low |
| **Etanercept vs ixekizumab vs placebo** | | | | | | | | |
| Griffiths et.al., 2015 (UNCOVER-2)^17^ | Low | Low | Low | Unclear | Low | Low | Low | Low |
| Griffiths et.al., 2015 (UNCOVER-3)^17^ | Low | Low | Low | Low | Low | Low | Low | Low |
| **Infliximab vs placebo** | | | | | | | | |
| Chaudhari et.al., 2001^18^ | Low | Low | Low | Low | Low | Unclear | Unclear | Low |
| Gottlieb et.al., 2004 (SPIRIT)^19^ | Low | Low | Low | Unclear | High | Unclear | Unclear | Low |
| Reich et.al., 2005 (EXPRESS)^20^ | Low | Low | Low | Low | Low | Unclear | Unclear | Low |
| Menter et.al., 2007 EXPRESS II)^21^ | Low | Low | Low | Low | Low | Unclear | Unclear | Low |
| Yang et.al., 2012^22^ | Unclear | Unclear | Unclear | Unclear | Low | Unclear | Unclear | Low |
| **Infliximab vs methotrexate** | | | | | | | | |
| Barker et.al., 2011 (RESTORE1)^23^ | Low | High | High | High | Unclear | Unclear | Unclear | Unclear |
| **Ixekizumab vs placebo** | | | | | | | | |
| Gordon et.al., 2016 (UNCOVER-1)^24^ | Low | Low | Low | Low | Low | Low | Low | Low |
| **Secukinumab 150 mg vs secukinumab 300 mg** | | | | | | | | |
| Mrowietz et.al., 2015 (SCULPTURE)^26^ | Unclear | Unclear | Low | Low | Low | Unclear | Unclear | Low |
| **Secukinumab 150 mg vs secukinumab 300 mg vs placebo** | | | | | | | | |
| Langley et.al., 2014 (ERASURE)^27^ | Low | Low | Low | Low | Low | Low | Low | Low |
| Blauvelt et.al., 2015 (FEATURE)^28^ | Low | Low | Low | Low | Low | Low | Low | Low |
| Paul et.al., 2015 (JUNCTURE)^29^ | Low | Low | Low | Low | Low | Low | Unclear | Low |
| **Ustekinumab vs placebo** | | | | | | | | |
| Tsai et.al., 2011 (PEARL)^30^ | Low | Low | Low | Unclear | Low | Unclear | Unclear | Low |
| Zhu et.al., 2013 (LOTUS)^31^ | Unclear | Unclear | Unclear | Unclear | Low | Low | Unclear | Low |
| Lebwohl et.al.a., 2015 (AMAGINE 2)^32^ | Low | Low | Low | Low | Low | Low | Low | Low |
| Lebwohl et.al.b., 2015 (AMAGINE 3)^32^ | Low | Low | Low | Low | Low | Low | Low | Low |
| **Ustekinumab 45 mg vc 90 mg vs placebo** | | | | | | | | |
| Leonardi et.al., 2008 ( PHOENIX 1)^33^ | Low | Low | Low | Low | Low | Unclear | Unclear | Low |
| Papp et.al., 2008 (PHOENIX 2)^34^ | Low | Low | Low | Unclear | Low | Unclear | Unclear | Low |
| Igarashi et.al, 2012^35^, NCT00723528, clinicaltrials.gov, 2014^36^ | Unclear | Unclear | Unclear | Unclear | Low | Unclear | Unclear | Low |
| **Etanercept vs ustekinumab 45 mg vs ustekinumab 90 mg** | | | | | | | | |
| Griffiths et.al., 2010 (ACCEPT)^37^ | Low | Low | Unclear | Low | Low | Low | Low | Low |
| **Etanercept vs ustekinumab vs no treatment** | | | | | | | | |
| Merck Sharp & Dohme 2015, NCT01276847, clinicaltrials.gov^38^ | Unclear | High | High | High | Low | Unclear | Unclear | Unclear |
| **Etanercept vs secukinumab 150 mg vs secukinumab 300 mg vs placebo** | | | | | | | | |
| Langley et.al., 2014 (FIXTURE)^27^ | Low | Low | Low | Low | Low | Low | Low | Low |

**Figure S1 Mantel*-*Haenszel risk difference of major adverse cardiovascular events in patients treated with (a) biologic therapies vs. placebo (b) tumour necrosis factor α inhibitors (TNFi) vs. placebo (c) Anti-interleukin (IL) -17A agents vs. placebo (d) ustekinumab vs. placebo**

**(a) Biologic therapies versus placebo**

**
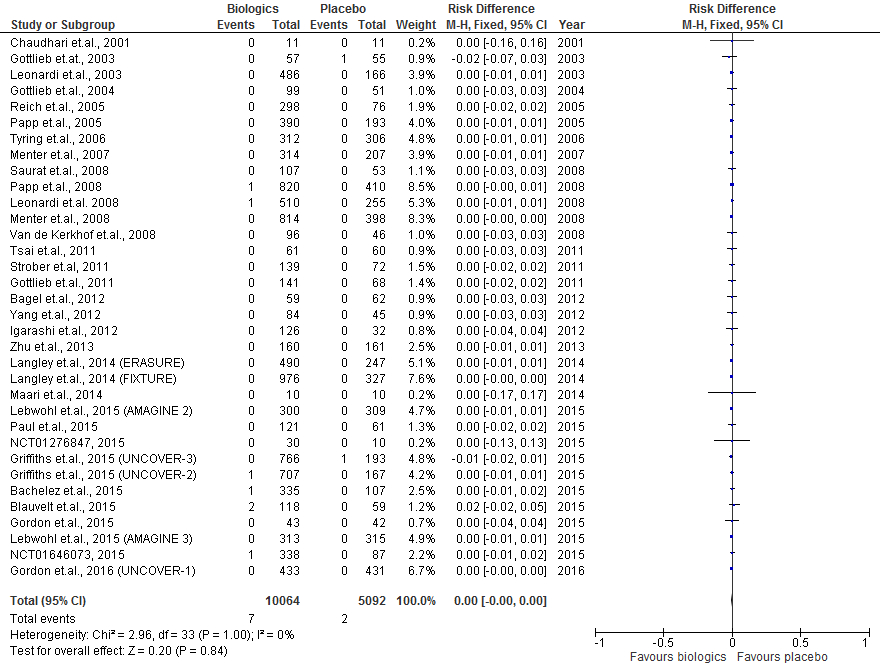
**

**(b) TNFi versus placebo**

**
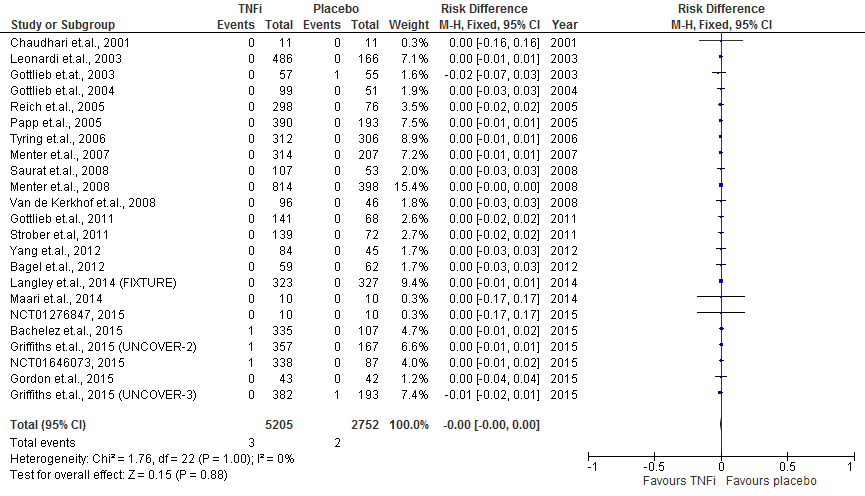
**

**(c) Anti-IL -17A agents versus placebo**

**
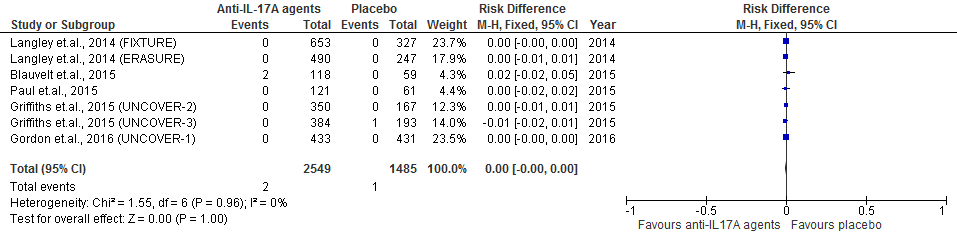
**

**(d) Ustekinumab versus placebo**


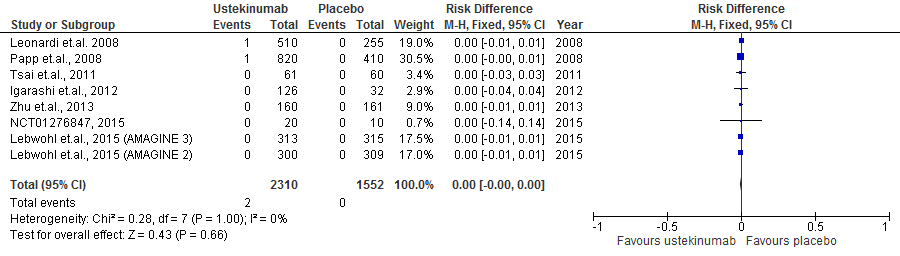


**References**

1 Menter A, Tyring SK, Gordon K, *et al.* Adalimumab therapy for moderate to severe psoriasis: A randomized, controlled phase III trial. *J Am Acad Dermatol* 2008; **58**:106–15.

2 Maari C, Bolduc C, Nigen S, *et al.* Effect of adalimumab on sleep parameters in patients with psoriasis and obstructive sleep apnea: A randomized controlled trial. *J Dermatolog Treat* 2014; **25**:57–60.

3 Gordon KB, Duffin KC, Bissonnette R, *et al.* A phase 2 trial of guselkumab versus adalimumab for plaque psoriasis. *N Engl J Med* 2015; **373**:136–44.

4 AbbVie. Safety and efficacy study of adalimumab in the treatment of plaque psoriasis. Available at: https://clinicaltrials.gov/show/NCT01646073 (last accessed on 17 January 2017).

5 AbbVie. Adalimumab M13-606 clinical study report R&D/13/997. Available at: https://www.abbvie.com/wp-content/uploads/2016/10/adalimumab-M13-606.pdf (last accessed on 17 October 2017).

6 Goldminz AM, Suarez-Farinas M, Wang AC, *et al.* CCL20 and IL22 Messenger RNA Expression After Adalimumab vs Methotrexate Treatment of Psoriasis: A Randomized Clinical Trial. *JAMA Dermatol* 2015; **151**:837–46.

7 Saurat JH, Stingl G, Dubertret L, *et al.* Efficacy and safety results from the randomized controlled comparative study of adalimumab vs. methotrexate vs. placebo in patients with psoriasis (CHAMPION). *Br J Dermatol* 2008; **158**:558–66.

8 Gottlieb AB, Matheson RT, Lowe N, *et al.* A randomized trial of etanercept as monotherapy for psoriasis. *Arch Dermatol* 2003; **139**:1627–32.

9 Tyring S, Gottlieb A, Papp K, *et al.* Etanercept and clinical outcomes, fatigue, and depression in psoriasis: double-blind placebo-controlled randomised phase III trial. *Lancet* 2006; **367**:29–35.

10 van de Kerkhof PCM, Segaert S, Lahfa M, *et al.* Once weekly administration of etanercept 50 mg is efficacious and well tolerated in patients with moderate-to-severe plaque psoriasis: a randomized controlled trial with open-label extension. *Br J Dermatol* 2008; **159**:1177–85.

11 Gottlieb A, Leonardi C, Kerdel F, *et al.* Efficacy and safety of briakinumab vs. etanercept and placebo in patients with moderate to severe chronic plaque psoriasis. *Br J Dermatol* 2011; **165**:652–60.

12 Strober BE, Crowley JJ, Yamauchi PS, *et al.* Efficacy and safety results from a phase III, randomized controlled trial comparing the safety and efficacy of briakinumab with etanercept and placebo in patients with moderate to severe chronic plaque psoriasis. *Br J Dermatol* 2011; **165**:661–8.

13 Bagel J, Lynde C, Tyring S, *et al.* Moderate to severe plaque psoriasis with scalp involvement: a randomized, double-blind, placebo-controlled study of etanercept. *J Am Acad Dermatol* 2012; **67**:86–92.

14 Bachelez H, van de Kerkhof PCM, Strohal R, *et al.* Tofacitinib versus etanercept or placebo in moderate-to-severe chronic plaque psoriasis: A phase 3 randomised non-inferiority trial. *Lancet* 2015; **386**:552–61.

15 Leonardi CL, Powers JL, Matheson RT, *et al.* Etanercept as monotherapy in patients with psoriasis. *N Engl J Med* 2003; **349**:2014–22.

16 Papp KA, Tyring S, Lahfa M, *et al.* A global phase III randomized controlled trial of etanercept in psoriasis: Safety, efficacy, and effect of dose reduction. *Br J Dermatol* 2005; **152**:1304–12.

17 Griffiths CEM, Reich K, Lebwohl M, *et al.* Comparison of ixekizumab with etanercept or placebo in moderate-to-severe psoriasis (UNCOVER-2 and UNCOVER-3): results from two phase 3 randomised trials. *Lancet* 2015; **386**:541–51.

18 Chaudhari U, Romano P, Mulcahy LD, *et al.* Efficacy and safety of infliximab monotherapy for plaque-type psoriasis: A randomised trial. *Lancet* 2001; **357**:1842–7.

19 Gottlieb AB, Evans R, Li S, *et al.* Infliximab induction therapy for patients with severe plaque-type psoriasis: A randomized, double-blind, placebo-controlled trial. *J Am Acad Dermatol* 2004; **51**:534–42.

20 Reich K, Nestle FO, Papp K, *et al.* Infliximab induction and maintenance therapy for moderate-to-severe psoriasis: A phase III, multicentre, double-blind trial. *Lancet* 2005; **366**:1367–74.

21 Menter A, Feldman SR, Weinstein GD, *et al.* A randomized comparison of continuous vs. intermittent infliximab maintenance regimens over 1 year in the treatment of moderate-to-severe plaque psoriasis. *J Am Acad Dermatol* 2007; **56**:31.e1–15.

22 Yang HZ, Wang K, Jin HZ, *et al.* Infliximab monotherapy for Chinese patients with moderate to severe plaque psoriasis: a randomized, double-blind, placebo-controlled multicenter trial. *Chin Med J* *(Engl)* 2012; **125**:1845–51.

23 Barker J, Hoffmann M, Wozel G, *et al.* Efficacy and safety of infliximab vs. methotrexate in patients with moderate-to-severe plaque psoriasis: results of an open-label, active-controlled, randomized trial (RESTORE1). *Br J Dermatol* 2011; **165**:1109–17.

24 Gordon KB, Blauvelt A, Papp KA, *et al.* Phase 3 trials of ixekizumab in moderate-to-severe plaque psoriasis. *N Engl J Med* 2016; **375**:345–56

25 Leonardi C, Blauvelt A, Langley RG, *et al.* Maintenance of efficacy among patients who achieve sPGA (0, 1): 60-week results from UNCOVER-1, a phase 3 trial of ixekizumab for moderate-to-severe plaque psoriasis. Presented at the 74^th^ *American Academy of Dermatology Annual Meeting*, Washington, D.C., U.S.A., 4–8 March 2016.

26 Mrowietz U, Leonardi CL, Girolomoni G, *et al.* Secukinumab retreatment-as-needed versus fixed-interval maintenance regimen for moderate to severe plaque psoriasis: A randomized, double-blind, noninferiority trial (SCULPTURE). *J Am Acad Dermatol* 2015; **73**:27–36.

27 Langley RG, Elewski BE, Lebwohl M, *et al.* Secukinumab in plaque psoriasis - results of two phase 3 trials. *N Engl J Med* 2014; 371:326–38.

28 Blauvelt A, Prinz JC, Gottlieb AB, *et al.* Secukinumab administration by pre-filled syringe: efficacy, safety and usability results from a randomized controlled trial in psoriasis (FEATURE). *Br J Dermatol* 2015; **172**:484–93.

29 Paul C, Lacour J-P, Tedremets L, *et al.* Efficacy, safety and usability of secukinumab administration by autoinjector/pen in psoriasis: a randomized, controlled trial (JUNCTURE). *J Eur Acad Dermatology Venereol* 2015; **29**:1082–90.

30 Tsai T-F, Ho J-C, Song M, *et al.* Efficacy and safety of ustekinumab for the treatment of moderate-to-severe psoriasis: a phase III, randomized, placebo-controlled trial in Taiwanese and Korean patients (PEARL). *J Dermatol Sci* 2011; **63**:154–63.

31 Zhu X, Zheng M, Song M, *et al.* Efficacy and safety of ustekinumab in Chinese patients with moderate to severe plaque-type psoriasis: results from a phase 3 clinical trial (LOTUS). *J Drugs Dermatol* 2013; **12**:166–74.

32 Lebwohl M, Strober B, Menter A, *et al.* Phase 3 studies comparing brodalumab with ustekinumab in psoriasis. *N Engl J Med* 2015; **373**:1318–28.

33 Leonardi CL, Kimball AB, Papp KA, *et al.* Efficacy and safety of ustekinumab, a human interleukin-12/23 monoclonal antibody, in patients with psoriasis: 76-week results from a randomised, double-blind, placebo-controlled trial (PHOENIX 1). *Lancet* 2008; **371**:1665–74.

34 Papp K a., Langley RG, Lebwohl M, *et al.* Efficacy and safety of ustekinumab, a human interleukin-12/23 monoclonal antibody, in patients with psoriasis: 52-week results from a randomised, double-blind, placebo-controlled trial (PHOENIX 2). *Lancet* 2008; **371**:1675–84.

35 Igarashi A, Kato T, Kato M, *et al.* Efficacy and safety of ustekinumab in Japanese patients with moderate-to-severe plaque-type psoriasis: Long-term results from a phase 2/3 clinical trial. *J Dermatol* 2012; **39**:242–52.

36 Janssen Pharmaceutical K.K. An efficacy and safety study of ustekinumab (CNTO 1275) in participants with plaque psoriasis. Available at: https://clinicaltrials.gov/ct2/show/results/NCT00723528 (last accessed on 17 January 2017).

37 Griffiths CEM, Strober BE, van de Kerkhof P, *et al.* Comparison of ustekinumab and etanercept for moderate-to-severe psoriasis. *N Engl J Med* 2010; **362**:118–28.

38 Merck Sharp & Dohme Corp. A study to assess the effect of ustekinumab (Stelara^®^) and etanercept (Enbrel^®^) in participants with moderate to severe psoriasis. Available at: https://clinicaltrials.gov/ct2/show/NCT01276847 (last accessed on 17 January 2017).
